# Closing gaps in the human genome using sequencing by synthesis

**DOI:** 10.1186/gb-2009-10-6-r60

**Published:** 2009-06-02

**Authors:** Manuel Garber, Michael C Zody, Harindra M Arachchi, Aaron Berlin, Sante Gnerre, Lisa M Green, Niall Lennon, Chad Nusbaum

**Affiliations:** 1 Genome Sequencing and Analysis Program, Broad Institute of MIT and Harvard, 7 Cambridge Center, Cambridge, MA 02142, USA; 2 Department of Medical Biochemistry and Microbiology, Uppsala University, SE-751 24 Uppsala, Sweden

## Abstract

The most recent release of the finished human genome contains 260 euchromatic gaps (excluding chromosome Y). Recent work has helped explain a large number of these unresolved regions as 'structural' in nature. Another class of gaps is likely to be refractory to clone-based approaches, and cannot be approached in ways previously described. We present an approach for closing these gaps using 454 sequencing. As a proof of principle, we closed all three remaining non-structural gaps in chromosome 15.

## Abstract 2

A novel method for closing non-structural gaps in the human genome assembly using 454 sequencing is presented here.

## Background

The finished sequence of human chromosome 15 contained nine sequence gaps at the time of publication [1]. Six of these gaps were flanked by segmental duplications, and recent work showed that one of these gaps could be closed by resolving assembly issues in segmental duplications [2]. A similar process has recently been undertaken genome-wide [3]. Three of the chromosome 15 gaps occur in regions of unique sequence with no evidence of copy number variation. Alignment of these regions to the publicly released Celera whole-genome shotgun assembly suggested gap sizes of 12, 10 and 9 kb [4], all substantially smaller than previous estimates [1]. No additional gap sequence was assembled in the more deeply covered HuRef assembly [5]. Further, flanks for two of these gaps could be aligned to scaffolds in the Rhesus macaque genome (rheMac2 assembly [6]), which provided size estimates similar to those from Celera. No clones spanned the orthologous regions in the chimpanzee genome assembly.

## Results

**Figure 1:**
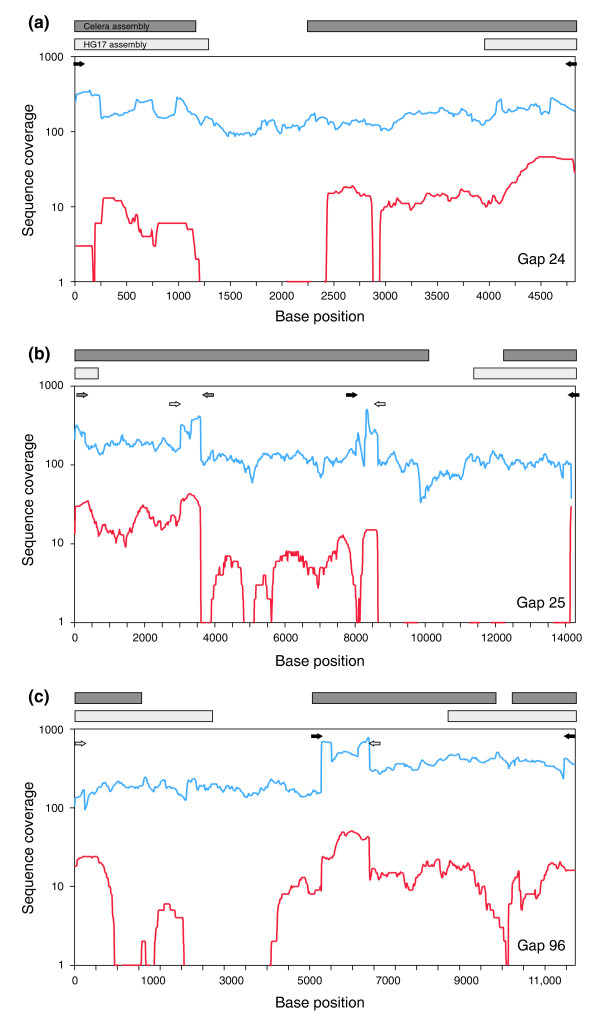
Coverage of gap regions. Sequence coverage of the gap regions on human chromosome 15 is shown for gaps at **(a) **24 Mb, **(b) **25 Mb and **(c) **96 Mb. The x-axis indicates base position in the local region containing each gap. The y-axis shows sequence coverage obtained in 454 reads (red line) and small insert library reads (blue line). Coverage was computed as the average in 10-base non-overlapping windows. Arrows indicate primers used to amplify the amplicons sequenced, color coded in pairs. Bars at top indicate bases present in the Celera (dark gray) and NCBI build 36 (light gray) assemblies.

Using both the NCBI build 36 and Celera assemblies we designed six primer pairs anchored in unique sequences that tiled the three gaps (for two of the three we used Celera contigs inside the build 36 gap to design primers; Table S1 in Additional data file 1). We amplified these regions via PCR from human genomic DNA (Coriell NA15510; see Materials and methods). End sequences of the PCR products matched the gap-flanking sequences, and product sizes on agarose gels closely matched the expected product sizes based on the gap sizing in the Celera assembly (Figure S1 in Additional data file 1). We then attempted to clone the PCR products directly, but sequencing of these showed that we were unsuccessful in obtaining clones that contained the desired product. Next, we produced and assembled small insert (average length 500 bp) 'shatter' libraries from the PCR products [7]. This approach of breaking difficult regions into much smaller fragments has been used with great success to resolve sequences challenging to the finishing process. These assemblies did provide new sequence extending into each of the three gaps (1,002 of 2,658 bp, 2,229 of 10,166 bp and 1,618 of 5,554 bp), but failed to yield the full sequence spanning any of the gaps (Figure 1).

As an alternative approach, we sheared and directly sequenced the gap-spanning PCR products using the 454 Life Sciences GS FLX [8]. Reads were assembled using a module of the ARACHNE assembler designed for 454 data [9] (see Materials and methods) since the 454 Newbler assembler does not assemble the sequences into a single contig. For each gap, the 454 reads were successfully assembled into a single, high-quality contig spanning the gap region (Figure 1). In one case, a clear second haplotype containing a 108-base deletion was manually assembled from the remaining data. Existence of this second haplotype was also supported by PCR product sizes on agarose gels (Figure S2 in Additional data file 1). In all three cases, the assembly was concordant with the expected region size (Figure S1 in Additional data file 1) and in perfect agreement with all AB 3730 sequence reads previously obtained from PCR product ends and shatter libraries. In the three closed gaps, the sequences obtained solely by 454 were 1.2 to 1.6 kb in length and primarily of low complexity (G+T rich on one strand) but without a clear motif or repeating unit (see below).

A key difference between the 454 methodology and traditional sequencing is that the 454 process has no bacterial cloning step. We theorized that previous failure to close the gaps was due to bias against cloning the gap sequence in standard *Escherichia coli *vector systems. To explore this, we designed PCR primers based on the complete 454 assembly and attempted to amplify and clone the regions that had been refractory to the initial clone-based approaches. These efforts yielded subclones; however, multiple lines of evidence demonstrated that all cloned products had deleted the majority of the low-sequence-complexity regions. Examination of capillary sequence traces from the subclones obtained from the PCR products showed that all sequenced subclones were missing the majority of the gap region and that deletions had clearly arisen at different locations in different subclones from each product. In all cases, alignment of the sequence showed the plasmid subclone insert to be 80 to 200 bases in length, while the actual lengths of the genomic sequences from which they were derived are all >1 kb. Finally, noise patterns in trace data from individual subclones suggested that deletion events had taken place during growth of the bacterial colony, resulting in a mixed population. Further, only two clones were found, both from the RPCI-11 Bacterial Artificial Chromosome (BAC) library, which spanned one gap and each had independently deleted the low complexity gap sequence along with a large surrounding region [2]. We posit that such regions are heavily underrepresented in large insert clone libraries because non-deleting clones containing the gap sequences appear to be non-viable We conclude from these results that the gap sequences are unstable or toxic when propagated in bacterial vectors, resulting in strong bias to remove them.

As part of the Human Genome Project (HGP) work, comprehensive efforts were made to close all gaps by clone-based methods [10]. All available end sequences from large insert clones were exhaustively analyzed in an attempt to identify clones spanning these gaps. In addition, all available large insert libraries (representing >50-fold physical coverage of the genome [10]) were probed by hybridization with probes designed from sequences flanking all gaps. Finally, all available primate whole genome sequence assemblies were examined in attempts to find orthologous sequences that could be used to make probes that were then used in hybridization screens. Only with the exhaustion of all of these methods did the HGP gap closure efforts cease. Thus, the gaps remained either because spanning clones could not be unambiguously identified, or because the intervening sequences did not propagate well in the cloning vectors used. We note that despite intense finishing efforts by the sequencing centres to capture all human gene models, two of the three gaps closed in chromosome 15 were located in intronic regions of gene loci (GABRA5 and GABRG3), and that there were also gaps in orthologous regions of all available primate genome assemblies. However, these recalcitrant regions remained uncaptured. We further confirmed that the Celera and HuRef assemblies also fail to span these regions with clones (S Levy, personal communication), and note that efforts by Bovee *et al. *[3] to screen additional Fosmid ends were unsuccessful in closing these gaps.

If the 454 methodology surmounts the problems posed by these regions, we might expect them to be represented in a whole genome shotgun sequence done by 454. We aligned the assembled gap regions to the recently released 454 reads from the Watson genome project [11] and found spotty coverage of two of the three gaps with reads landing within our finished sequence. This indicates that the 454 whole genome shotgun was able to represent these sequences, but failed to completely cover the gaps.

Analysis of the gap sequences that were recalcitrant to cloning showed they were largely composed of low complexity sequence, highly enriched in G+T on one strand, C+A on the other. To estimate how rare this type of sequence is, we slid a 4 kb window across the genome and measured G+T or C+A content. Using the empirical distribution obtained from these measurements, we computed the probability of picking a 4 kb region by chance with G+T or C+A content as high as any of our three gap regions at *P *= 0.0002; the likelihood of picking three such regions by chance is therefore extremely low (*P *= 8 × 10^-12^). We find, however, that regions (431 genome-wide) with even longer stretches of this type of sequence are present in the finished human genome sequence and captured in large insert clones. We conclude that sequence composition plays a significant role in what makes these regions difficult to clone, but there are likely to be other factors as well.

It has been shown that sequence rich in alternating pyrimidimes/purines tends to adopt Z-DNA conformations and that such DNA conformation tends to be difficult to clone [12,13]. In all three cases the sequence recalcitrant to cloning harbored large stretches (271, 485 and 364 bases long, respectively) of alternating pyrimidines/purines of the type that tends to form Z-DNA structure as postulated in Konopka *et al. *[12]. Our genome-wide analysis again revealed longer stretches within finished clones; however, since it is highly unlikely to find three such stretches by mere chance (*P *= 10^-9^), we conclude that the tendency of these regions to adopt Z-DNA conformation is likely, along with sequence composition, to also contribute to the intolerance of the gap sequences to cloning in *E. coli *vectors.

Having closed three gaps on chromosome 15, we sought to determine how many other gaps in the genome might be closable by this method. In NCBI human build 36 there are 260 remaining gaps (excluding chromosome Y and the 29 gaps that contain heterochromatic regions, including centromeres and acrocentric short arms). We carried out a similar analysis to Eichler *et al*. [14], showing that the gaps fall into three classes as defined by sequence composition of the region. Type I gaps are subtelomeric: there are nine gaps in subtelomeric regions containing telomere-associated repeats. Type II contain duplicated euchromatin; this includes 30 pericentromeric gaps (within 1 megabase of a centromere) and 94 gaps flanked by segmental duplications. Type III gaps are in unique euchromatin; these 127 gaps do not show signatures of duplication or structural polymorphism. Based on our work here, we propose they remain gaps because they contain sequences recalcitrant to the standard bacterial cloning methods used for libraries in the HGP.

Class I and II gaps are likely to arise from unresolved structural complexity in the genome and can be attacked by the methodology of carefully reassembling existing tiling paths or by reassembling the area using a single haplotype, as described previously [2,3]. The three gaps closed here are representative of class III; the gaps that appear to remain because they contain sequences that are grossly underrepresented or deleted in clone libraries and therefore are likely to be refractory to cloning techniques used in the HGP. Our data suggest that these sequence regions are relatively small, contrary to their sizing in the clone-based assembly, and thus amenable to standard PCR amplification and sequencing by 454 (see Additional data file 2 for size estimations based on other human and primate assemblies).

## Discussion

We have demonstrated a simple and scalable method for finishing non-structural gaps in genome assemblies. While clone-based methods remain an effective means of attacking structural gaps, they will not resolve gaps that arise from sequences recalcitrant to bacterial cloning. Previous reports suggest that some of these gaps can be captured by extreme methods, including cloning in yeast [13,15], but these methods are laborious and less scalable. It is possible that 454 whole genome shotgun would close some of them. However, alignment of 10 kb of sequence flanking all class III gaps to the Watson contigs obtained from reads that did not map to the NCBI build 36 assembly [11] resulted in only one contig going 800 bp into the gap and no contigs that spanned gaps. We reason that type III gaps may be underrepresented in the whole genome sequence because these low complexity sequences may be partially underrepresented in the 454 library construction, and will generate poorer quality data and be more difficult to align or assemble when not specifically targeted.

The human genome still contains 127 class III gaps, 21 of which overlap RefSeq gene annotations [16]. Most of these will have been abandoned as refractory to current techniques because of the absence of spanning large insert clones. In most cases, PCR would not have been attempted, given the estimated sizes of the gaps (44, 101, and 22 kb for the three closed here); if it had, most likely the poor quality bands given by standard PCR or the resulting deletions in cloned PCR products would have convinced finishers that the PCR was not accurate. However, given the great effort already carried out to close them, we expect that flanking clones will be very close to the refractory region and, therefore, many gaps will be small (Additional data file 2) and likely to be finishable by the method described here. The technique we present could also be applied to the targeted closure of gaps in other finished or near finished genomes such as mouse and dog [17,18], which contain 103 (D Church, personal communication) and 47 (K Lindblad-Toh, personal communication) class III gaps, respectively.

## Materials and methods

### DNA resources

All sequenced PCR products were generated from genomic DNA from the NA15510 cell line. This cell line was the DNA source for a Fosmid library (WIBR-2) that was used in completing the HGP. All products were also successfully amplified from genomic DNA from other individuals, cell lines NA12003, NA18489 and NA18547 (data not shown). Samples were obtained from the Corriell Institute for Medical Research [19].

### Assembly

A specialized version of the ARACHNE assembler was used to create *de novo *assemblies of 454 data [9,20]. This version of the algorithm takes advantage of both the deep sequence coverage we used and the small sizes of the regions assembled, which allowed us to assume that no sequence repeat would be large enough to fully contain an average read. It also relaxes the dependence on read pairing, as paired 454 reads were not used. The assembly process consists of two steps. First, an initial pre-processing stage is run, the output of which is an initial read layout. This is identical to the process employed in the published ARACHNE algorithm. This stage generally results in a fragmented assembly. Second, we employ an iterative procedure that incrementally merges contigs and improves read placement. This procedure works by: aggressively merging the existing contigs, which is safe where sequence repeats are smaller than the average read size; identifying 'suspect regions,' defined as sites in the assembly where errors are likely (small repeat units that may be misassembled and regions where coverage drops below 10% of the average assembly coverage); removing reads from the assembly that are in suspect regions; and attempting to relocate reads removed from suspicious regions to better placements in the assembly. Reads that can be aligned uniquely and with a good alignment score (as defined in the standard ARACHNE assembler [9,20]) are 're-placed' in the assembly. The version of the assembler used in this work is freely and publicly available [20].

Electrophoresis gel sizing of gap 24 (Figure S2 in Additional data file 1) showed the presence of a 108 base polymorphism. Manual inspection of the assembled region allowed us to identify shatter reads corresponding to both haplotypes and thus to define the breakpoints of the insertion/deletion polymorphism.

### Molecular biology methods

Primers (Table S1 in Additional data file 1) were designed using Primer3 [21] based on the genome assembly that provided most sequence into the gap. Primers for gaps 24, 25 and the right flank of gap 96 were picked from the Celera assembly. The left flanking primer from gap 96 was picked from the HGP assembly. In all cases additional primers were designed to PCR amplify regions only covered by the Celera assembly. Where multiple PCR products were generated for a single gap, they were pooled for 454 library construction.

PCR reactions were carried out in a 10 μl volume containing 15 ng of template DNA, primers (0.2 μM each), dNTPs (0.5 mM each) and 2.5 units Herculase polymerase in 10× Herculase reaction buffer (Stratagene, La Jolla, CA, USA). The amplification was carried out with an initial treatment at 94°C for 2 minutes, followed by 30 cycles of 94°C for 30 s, 51°C for 30 s and 68°C for 10 minutes.

PCR products were cloned by ligation into either the pCR^® ^2.1-TOPO^® ^or pCR^® ^II-TOPO^® ^vector (Invitrogen, Carlsbad, CA, USA), as directed by the manufacturer. Small insert subclone libraries were made in the pUC19 (New England BioLabs, Ipswitch, MA, USA) vector using the published method [7].

All Sanger chemistry sequencing (PCR product end sequencing, cloned PCR products and small insert libraries) was carried out using BigDye version 3.1 reagents on ABI 3730xl instruments (Applied Biosystems, Foster City, CA, USA). 454 sequencing was carried out as recommended by the manufacturer on GS-20 FLX instruments (454 Life Sciences, Branford, CT, USA). Sequencing runs generated a total of 32,830 reads, of which 76% (24,875 reads), averaging 209 bases, were incorporated in the assemblies. Sequencing reads were deposited in the NCBI short read archive [22], under project identification numbers [SRP000644] and [SRP000645].

Nucleotide sequences corresponding to the three closed gaps have been deposited in GenBank with accession numbers [GenBank:EU606048], [GenBank:EU606049], and [GenBank:EU606050]. The assembled shorter haplotype of gap I is submitted separately as [GenBank:EU606051].

## Abbreviations

HGP: Human Genome Project.

## Authors' contributions

MG, MCZ and CN conceived the project and wrote the manuscript, MG and MCZ carried out data analysis, LMG and NL carried out molecular biology experiments, HMA and AB performed assembly and finishing work, and SG contributed the modified ARACHNE assembler used to put together the sequenced data. All authors approved the final manuscript.

## Additional data files

The following additional data are available with the online version of this paper: Table S1, providing gap region information, Table S2 listing the clones flanking the gaps in HGP NCBI build 36, and Figures S1 and S2, showing gel electrophoresis images with approximate amplified region sizes (Additional data file 1); a table listing the sizes of type III gaps estimated from aligning flanks to primate assemblies (Additional data file 2).

## Supplementary Material

Additional data file 1 Tables S1: gap region information, including location, name, primers used to amplify sequence, original (HGP) size, and size after closing. Tables S2: clones flanking the gaps in HGP NCBI build 36. Figures S1 and S2 show gel electrophoresis images with approximate amplified region sizes. Click here for file

Additional data file 2 New clones that closed type III gaps as reported in Bovee *et al. *[3] are shown in bold italics. Click here for file
