# Supplementary material for: Closing gaps in the human genome using sequencing by synthesis
Source: Genome Biol. 2009 Jun 2;10(6):R60. doi: 10.1186/gb-2009-10-6-r60 (PMC2718494; doi:10.1186/gb-2009-10-6-r60)
Supplement: Additional data file 1 — Tables S1: gap region information, including location, name, primers used to amplify sequence, original (HGP) size, and size after closing. Tables S2: clones flanking the gaps in HGP NCBI build 36. Figures S1 and S2 show gel electrophoresis images with approximate amplified region sizes. [file gb-2009-10-6-r60-S1.doc]

**Supplementary Information.**

# Table S1. Primer pairs used to amplify regions.

# Primer pairs used to amplify gap regions not contained in the HGP. Gap positions (in megabases) are given on NCBI human build 36 along with their original estimate and the actual sequence added by the assembly of the 454 data. (*)Note: The assembly used to estimate the amount of sequence added to gap24 was based on the modified assembly described in [1] (see also table S2 below for the flanking clones used).

| **GAP** | **Primer**  **name** | **Left primer sequence** | **Right primer sequence** | **Original build 36 size** | **Size after closing** |
| --- | --- | --- | --- | --- | --- |
| Gap 24 (24.7 Mb) | Gap24 | GAGCTCCCTTGTCCTGATTTAACTTGAATTG | AGGAAACTGAGGGGAAACTGGATTTGAC | 44Kb | 1972 bases^*^ |
| Gap 25 (25.2 Mb) | Gap25_1 | AGCTTCTTCAAACTACTGAAGAGTTAGCCAGGTC | GGCTTGGTATGCTTGATGTTGGTATGC | 100Kb | 10485 bases |
|  | Gap25_2 | ATGTGGAAGGCTGTACTTCCAGAGAAACC | TTCTGTTATCCTAACTAAACAACAGAGAGATGCCC |  |  |
|  | Gap25_3 | TGAATTTCCCAGTAGCCAGAGAACAAGG | TCCATTTGAGGTTACAGACTAAAGATCACTAAGCAG |  |  |
| Gap 96 (96.3 Mb) | Gap96_1 | GTTCACCTGGATACAATGGAGCATTAGACTG | TGGTCAAAGGCAGTTAAATAGAAGAGAACTTTCAG | 22Kb | 3978 bases |

#
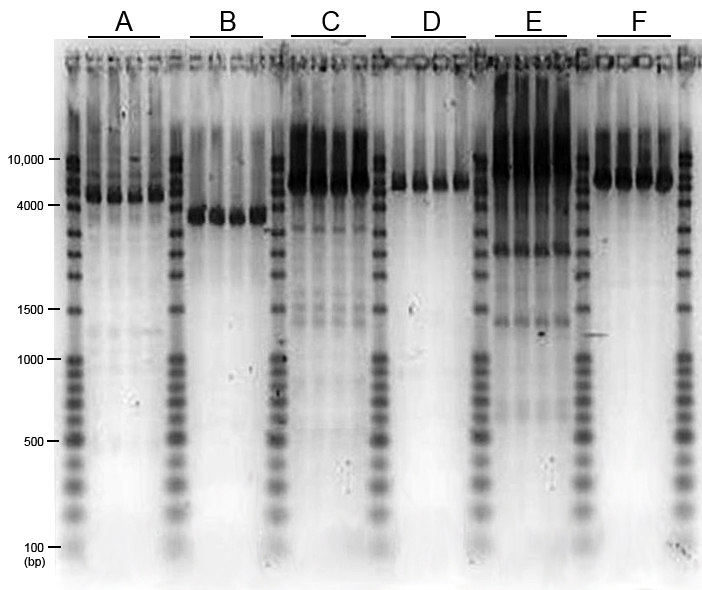


# Figure S1. Gel electrophoresis showing approximate primer product sizes.

Each lane corresponds to each of the 6 primer pairs used (see table S1). A: gap24 , B: gap25_1, C: gap25_2, D: gap25_3, E: gap96_1 F: Gap96_2. All amplified products were sized between 3.5 and 8 kb.


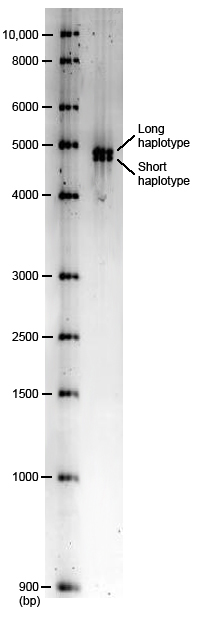


# Figure S2. Gel electrophoresis showing the 108 base polymorphism in gap 24.

# The NA15510 cell line is heterozygous for a length polymorphism which is clearly visible in this gel electrophoresis of the amplicons obtained by PCR using the gap I primers.

**Gap Classification.**

For this study we focused on gaps in the human genome sequence that were non-structural in nature. We define non-structural gaps as those that are not a) *centromeric*, annotated as containing a centromere in the AGP; b) *acrocentric*, annotated as a heterochromatic short arm in the AGP; c) *associated with duplication*, 5 kb of a segmental duplication, or within one megabase of a centromere or telomere. Our analysis identified 127 nonstructural gaps in HGP build 36.

**Chromosome 15 accessions and AGP tiling path**

Table S2. Tiling path for closed gaps

| **Gap** | **Left-flanking accession** | **Left-flanking clone name** | **Right-flanking accession** | **Right-flanking clone name** |
| --- | --- | --- | --- | --- |
| Gap 24 | AC145196 | WI2-83747H10 | AC145167 | XXfos-87138G1 |
| Gap 25 | AC145436 | WI2-86698B3 | AC144833 | WI2-82651E9 |
| Gap 96 | AC144834 | WI2-802163C5 | AC144835 | WI2-83372E10 |

1. Zody, M.C. et al: **Analysis of the DNA sequence and duplication history of human chromosome 15.** *Nature* 2006, **440**:671-5
